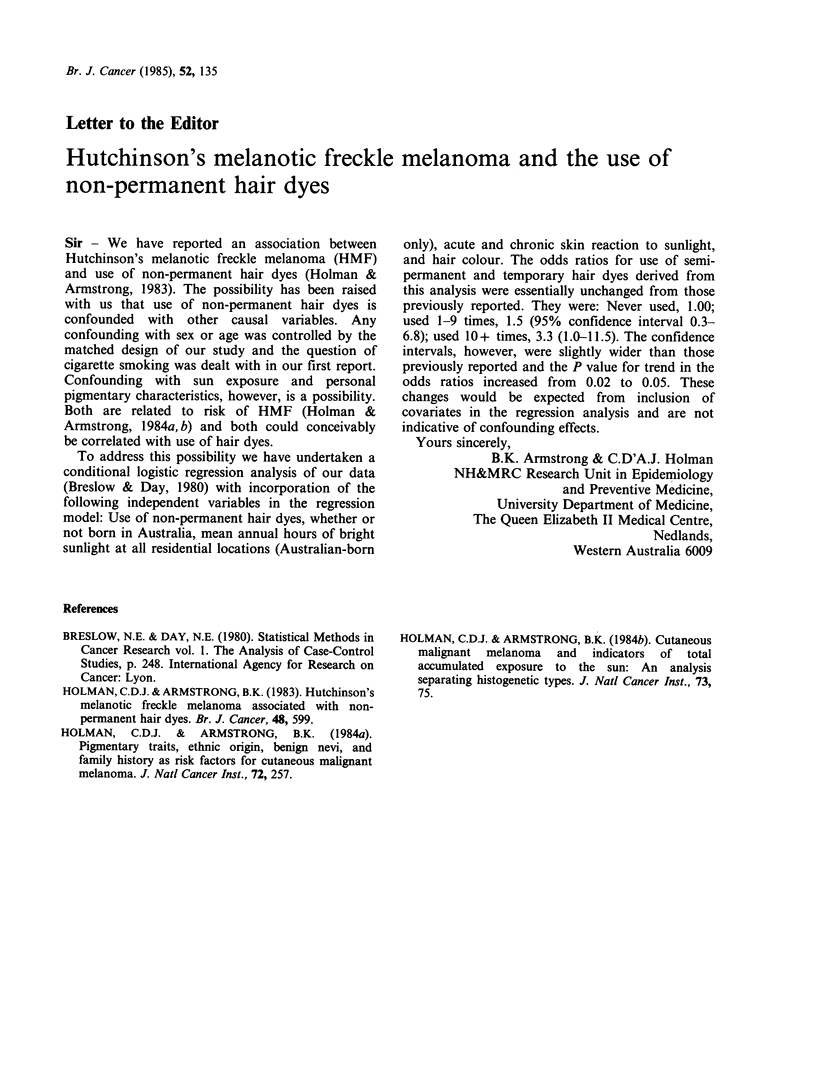# Hutchinson's melanotic freckle melanoma and the use of non-permanent hair dyes.

**DOI:** 10.1038/bjc.1985.162

**Published:** 1985-07

**Authors:** B. K. Armstrong, C. D. Holman


*Br. J. Cancer* (1985), **52**, 135

### Letter to the Editor

### Hutchinson's melanotic freckle melanoma and the use of non-permanent hair dyes

**Sir** – We have reported an association between Hutchinson's melanotic freckle melanoma (HMF) and use of non-permanent hair dyes (Holman & Armstrong, 1983). The possibility has been raised with us that use of non-permanent hair dyes is confounded with other causal variables. Any confounding with sex or age was controlled by the matched design of our study and the question of cigarette smoking was dealt with in our first report. Confounding with sun exposure and personal pigmentary characteristics, however, is a possibility. Both are related to risk of HMF (Holman & Armstrong, 1984*a, b*) and both could conceivably be correlated with use of hair dyes.

To address this possibility we have undertaken a conditional logistic regression analysis of our data (Breslow & Day, 1980) with incorporation of the following independent variables in the regression model: Use of non-permanent hair dyes, whether or not born in Australia, mean annual hours of bright sunlight at all residential locations (Australian-born only), acute and chronic skin reaction to sunlight, and hair colour. The odds ratios for use of semi-permanent and temporary hair dyes derived from this analysis were essentially unchanged from those previously reported. They were: Never used, 1.00; used 1–9 times, 1.5 (95% confidence interval 0.3–6.8); used 10+ times, 3.3 (1.0–11.5). The confidence intervals, however, were slightly wider than those previously reported and the *P* value for trend in the odds ratios increased from 0.02 to 0.05. These changes would be expected from inclusion of covariates in the regression analysis and are not indicative of confounding effects.

Yours sincerely,

B.K. Armstrong & C.D'A.J. Holman
NH&MRC Research Unit in Epidemiology
and Preventive Medicine,
University Department of Medicine,
The Queen Elizabeth II Medical Centre,
Nedlands,
Western Australia 6009

### References

BRESLOW, N.E. & DAY, N.E. (1980). Statistical Methods in Cancer Research vol. 1. The Analysis of Case-Control Studies, p. 248. International Agency for Research on Cancer: Lyon.

HOLMAN, C.D.J. & ARMSTRONG, B.K. (1983). Hutchinson's melanotic freckle melanoma associated with non-permanent hair dyes. *Br. J. Cancer*, **48**, 599.

HOLMAN, C.D.J. & ARMSTRONG, B.K. (1984*a*). Pigmentary traits, ethnic origin, benign nevi, and family history as risk factors for cutaneous malignant melanoma. *J. Natl Cancer Inst.*, **72**, 257.

HOLMAN, C.D.J. & ARMSTRONG, B.K. (1984*b*). Cutaneous malignant melanoma and indicators of total accumulated exposure to the sun: An analysis separating histogenetic types. *J. Natl Cancer Inst.*, **73**, 75.